# Preliminary Evidence for Increased Histone Succinylation as a Potential Epigenetic Marker for Longevity

**DOI:** 10.1111/acel.70346

**Published:** 2025-12-23

**Authors:** Stephanie Stransky, Sarah Graff, Kai Mao, Derek M. Huffman, Sofiya Milman, Nir Barzilai, Simone Sidoli

**Affiliations:** ^1^ Department of Biochemistry Albert Einstein College of Medicine New York New York USA; ^2^ Department of Molecular Pharmacology Albert Einstein College of Medicine New York New York USA; ^3^ Institute for Aging Research, Department of Medicine Albert Einstein College of Medicine New York New York USA; ^4^ Department of Genetics Albert Einstein College of Medicine New York New York USA

**Keywords:** aging, chromatin modifications, epigenetics, healthspan, histone succinylation, longevity, progeny of long‐lived individuals

## Abstract

Histone post‐translational modifications (PTMs) are critical regulators of chromatin structure and gene expression, with broad implications for development, metabolism, and aging. While canonical modifications such as methylation and acetylation are well characterized, the role of histone succinylation remains poorly understood. Here, we investigated histone succinylation in the context of aging and exceptional longevity. Using mass spectrometry–based proteomics, we quantified histone succinylation in B‐cells from four groups: young individuals, older individuals without parental longevity (OPUS), long‐lived individuals, and offspring of long‐lived individuals (OPEL). We found that histone succinylation was significantly elevated in the OPEL group compared to both young and OPUS cohorts. Nuclear proteomics further revealed enrichment of succinylated proteins in OPEL samples, supporting a role for succinylation in chromatin organization. To test whether succinate availability impacts healthspan, we supplemented middle‐aged mice with succinic acid. While body weight, frailty index, and cognition were unaffected, succinic acid improved motor coordination and muscle strength. Together, our findings provide preliminary evidence that enhanced histone succinylation may serve as a protective epigenetic mechanism in individuals predisposed to exceptional longevity, and that succinate supplementation can selectively improve aspects of physical performance during aging.

## Graphical abstract

Elevated histone succinylation distinguishes offspring of long‐lived individuals and aligns with enhanced nuclear functions, while succinate supplementation selectively improves motor performance in aging mice, suggesting succinylation as a candidate protective epigenetic feature of longevity.

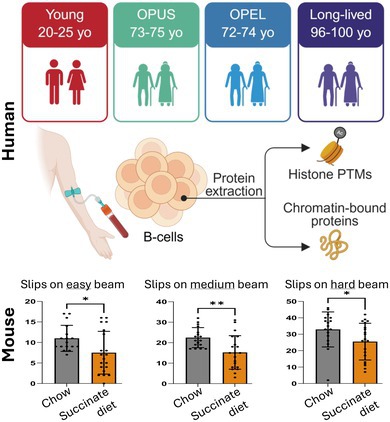

1

Aging is a complex biological process marked by a progressive decline in cellular and tissue function, increasing susceptibility to cancer, cardiovascular disease, and neurodegenerative disorders (Guo et al. 2022). Among the molecular hallmarks of aging, epigenetic alterations are thought to play a pivotal role in mediating these age‐related phenotypic changes (López‐Otín et al. 2023). Histone post‐translational modifications (PTMs) are recognized as key players in chromatin dynamics, gene regulation, and cellular identity. While canonical modifications such as methylation and acetylation have been extensively studied, growing evidence indicates that less common PTMs also contribute to aging regulation. One such modification, histone succinylation, first described in 2012 (Zhang et al. 2011), remains poorly characterized but may provide critical insights into the molecular basis of longevity.

Histone succinylation involves covalent attachment of a succinyl group to lysine residues, introducing a bulky negative charge that alters nucleosome structure and chromatin accessibility (Liu et al. 2021; Smestad et al. 2018; Zorro Shahidian et al. 2021). Unlike more prevalent modifications such as acetylation, succinylation is relatively uncommon, and its biological implications are just beginning to emerge. Recent studies have implicated protein succinylation in various cellular processes, including metabolism, stress response, and mitochondrial function (Yang and Gibson 2019; Weyh et al. 2024), all of which are intrinsically linked to aging (Amorim et al. 2022). Because succinylation depends on succinyl‐CoA, a tricarboxylic acid (TCA) cycle intermediate, it represents a direct interface between cellular metabolism and epigenetic regulation (Smestad et al. 2018; Trefely et al. 2020). This metabolic–epigenetic connection makes histone succinylation a compelling candidate for studies of chromatin regulation in aging and exceptional longevity.

One of the critical challenges in understanding the role of histone succinylation lies in its frequent co‐occurrence with acetylation at the same genomic loci, making it difficult to parse out the specific functions of each modification (Smestad et al. 2018). Some studies have suggested that succinylation and acetylation have similar activating roles in transcription (Smestad et al. 2018; Zorro Shahidian et al. 2021; Li et al. 2023), although the chemical properties and steric hindrance of a succinyl group compared to an acetyl group would suggest that the biology is likely more complex.

Offspring of long‐lived individuals (OPEL), represent a unique population for studying the molecular basis of longevity (Barzilai et al. 2003; Atzmon et al. 2005; Atzmon et al. 2004). Despite being in their 70s, these individuals often display remarkably lower rates of age‐related diseases, such as cardiovascular disease, cancer, and neurodegenerative disorders, compared to the general population (Adams et al. 2008; Galioto et al. 2008). Direct evidence that the OPEL cohort is more protected from cardiovascular diseases than the OPUS group was published by Gubbi et al. (2017). The genetic and epigenetic factors that contribute to this exceptional healthspan remain poorly understood, but the OPEL cohort offers an unprecedented opportunity to investigate potential protective mechanisms that promote healthy aging. Our study aims to quantitatively assess the levels of histone succinylation in a cohort of healthy donors divided among young (20s), older individuals with a projected usual longevity (70s, OPUS group), long‐lived individuals (> 95) and older progeny of long‐lived individuals (70s, OPEL group) (Figure 1a). Specifically, we isolated B‐ cells from 20 donors (5 per group) using FACS sorting. Details on the ages, sex and groups of the donors are listed in Table S1. Following cell isolation, histone proteins were extracted and subjected to mass spectrometry‐based proteomics to quantify succinylation levels on specific histone residues. We have identified significant differences in the levels of histone succinylation between the OPEL cohort and the young cohort (Figure 1b,c).

**FIGURE 1:**
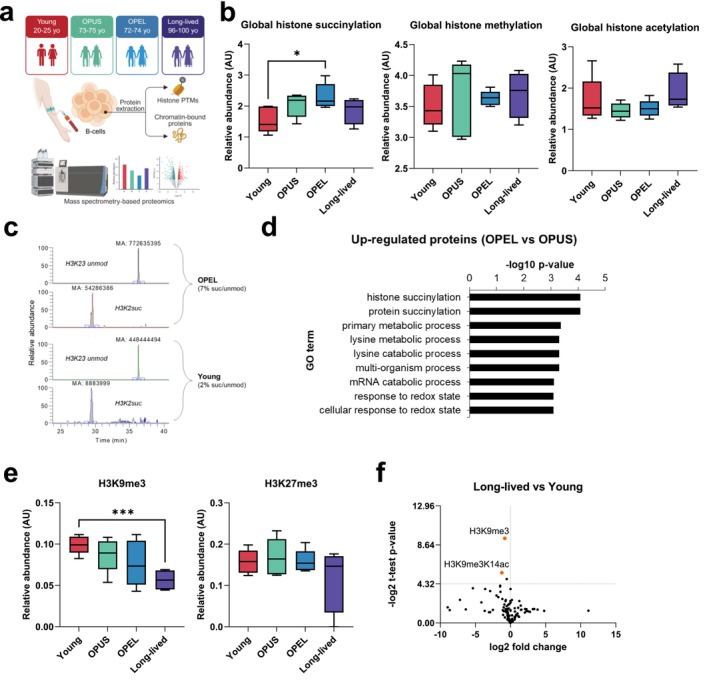
Histone succinylation in aged groups. (a) Workflow. B‐cells are purified from four groups of donors, namely young individuals (20s), older individuals (OPUS, 70s), the progeny of long‐lived individuals (OPEL, 70s), and long‐lived individuals (95 and older). (b) Quantification of global histone succinylation (left), methylation (center) and acetylation (right) using mass spectrometry. The relative abundance is calculated by summing the intensities of all peptides identified with the given modification vs. all peptide intensities. (c) Extracted ion chromatograms of the peptide containing H3K23succinyl to manually validate the detection in mass spectrometry of succinylated peptides. (d) Gene Ontology enrichment of proteins quantified via mass spectrometry from the nuclear proteome of the OPEL versus the OPUS group. Functional annotation was obtained using GOrilla (Eden et al. 2009). (e) Quantification of total levels of H3K9me3 (left) and H3K27me3 (right) with mass spectrometry. The relative abundance was obtained by summing all peptides containing the given modification versus the intensity of all (un)modified peptides sharing the same sequence. (f) Volcano plot displaying the quantified peptides in long‐lived individuals vs. young group. Highlighted are the two peptides quantified to be modified with H3K9me3.

In parallel, we isolated the nuclear proteome for each sample to perform proteomics of the soluble nuclear proteins. By comparing the Gene Ontology annotations of proteins enriched in the OPEL group compared to the OPUS group, we found that protein succinylation was one of the most enriched terms (Figure 1d). Albeit these are still preliminary data, our results suggest that histone succinylation is worth exploring as potential marker of “protected” chromatin, impacting chromatin accessibility and gene transcription. The full list of identified proteins is available as Table S2.

Based on general consensus, aging is associated with a general loss of heterochromatin markers (Tsurumi and Li 2012; Villeponteau 1997). By using the cohort available to us, we decided to extract abundances of the two major silencing histone marks; histone H3 K9me3 and K27me3, to verify the quality of our analysis and confirm that this pattern is reproduced in our cohort as well. Indeed, we quantified a monotonic decrease of both marks from the young group to the group of long‐lived individuals (Figure 1e). Notably, H3K9me3 was the most significantly downregulated. Given that our mass spectrometry‐based histone proteomic techniques allow us to quantify peptides that contain co‐existing modifications, we were able to demonstrate that both histones, modified only with H3K9me3 as well as modified with the co‐occuring mark H3K9me3K14ac, were reduced in long‐lived individuals compared to the young cohort (Figure 1f). This suggests that silencing marks are overall less abundant in older individuals regardless of the combinatorial histone code where they are catalyzed.

By leveraging the identified nuclear proteome, we quantified proteins involved in chromatin organization and gene expression in the samples from the long‐lived individuals group (Figure 2a,b), suggesting enhanced nuclear processes linked to genome maintenance and transcriptional control. To investigate whether these changes contribute to improve healthspan, mice at 18 months of age, were switched from a normal chow to a succinic acid diet and then monitored for 8 weeks. Physiological and behavioral assessments showed that a succinic acid diet did not significantly alter body weight (Figure 2c), indicating no effect on overall growth. Similarly, there was no change in the frailty index (Figure 2d), suggesting that the diet did not broadly influence age‐related health deficits. In the box maze test (Figure 2e), both groups improved with training, reflecting intact learning and memory processes, with no clear advantage for the succininc acid group. In contrast, mice fed with succinic acid displayed improved motor coordination and balance in the balance beam test, as shown by fewer slips across easy, medium, and hard beams (Figure 2f). Furthermore, neuromuscular strength was enhanced in succinic acid‐fed mice, as evidenced by longer grip retention times in the grip strength test (Figure 2g).

**FIGURE 2:**
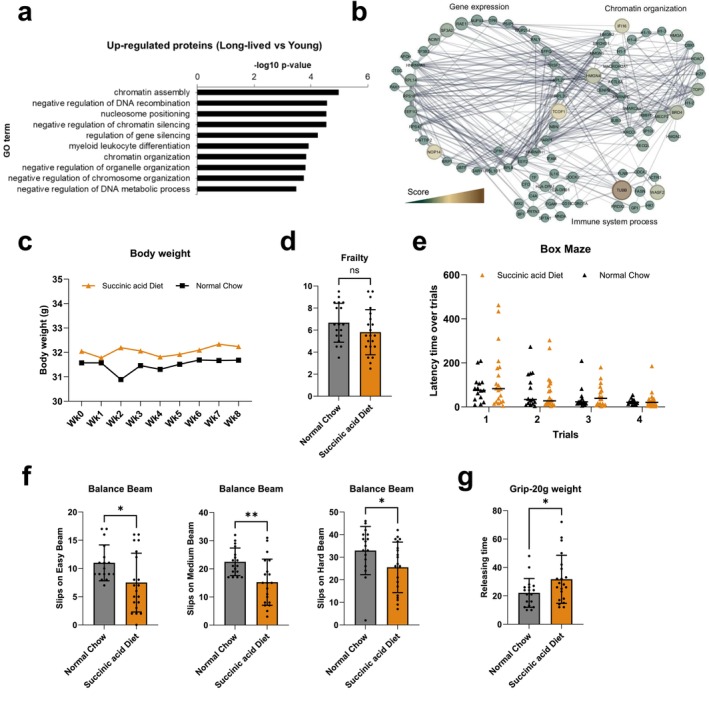
Effects of succinylation. (a) Gene Ontology enrichment of proteins quantified via mass spectrometry from the nuclear proteome of the long‐lived individuals hvs the young group. (b) Protein–protein interaction network of upregulated proteins in the soluble nuclear fraction of long‐lived individuals. Size of nodes represents *p‐value*, color darkness represents the score (fold change enrichment times the *p‐*value), and line thickness represents the score of interaction confidence retrieved from the software String. The network was constructed by Cytoscape (Shannon et al. 2003). (c) Body weight measured over 8 weeks. (d) Frailty index (general age‐related health deficits). (e) Box maze performance. (f) Balance beam performance on easy (left), medium (center) and hard (right) beams. (g) Grip strength test (20 g weight). Data are shown as mean ± SD.

Overall, our data suggest that succinylation may act as a protective epigenetic mechanism, particularly in the progeny of long‐lived individuals, who are projected to have extended healthspans compared to the general population. The enrichment of succinylation could indicate a greater capacity for chromatin compaction, preventing the transcriptional dysregulation and genomic instability typically associated with aging. This hypothesis aligns with previous work demonstrating that chromatin organization is a key factor in aging (Tsurumi and Li 2012; Feser and Tyler 2011), where the loss of heterochromatin can lead to global gene activation, increased DNA damage, and cellular senescence (Sun et al. 2018). Our findings also suggest that while succinic acid supplementation (even if treatment was relatively short term) does not broadly impact body weight, frailty, or cognition, it may confer benefits for motor function and muscle strength. However, it is important to emphasize that the results from our human cohort are severely limited by the low number of participants in this study, and that these findings are only preliminary results toward understanding the full scope of histone succinylation in the aging process. The precise molecular pathways through which succinylation regulates chromatin structure and gene expression, and whether these changes directly contribute to the extended healthspan, require further investigation.

## Author Contributions

Stephanie Stransky and Sarah Graff performed experiments, analyzed data, and contributed to figure preparation; they contributed equally to this work. Kai Mao and Derek M. Huffman conducted the mouse healthspan studies and analyzed the corresponding data. Sofiya Milman and Nir Barzilai recruited and characterized the human cohorts and provided clinical expertise on exceptional longevity. Simone Sidoli conceived and supervised the study, secured funding, and wrote the manuscript with input from all authors.

## Funding

This work was supported by NIH Office of the Director, S10OD030286. Hevolution Foundation (AFAR). National Institutes of Health (NIH), R01AG061155, P30AG038072.

## Conflicts of Interest

The authors declare no conflicts of interest.

## Supporting information


**Data S1:** acel70346‐sup‐0001‐Supinfo.docx.

**Table S1:** Information of the donor cohorts analyzed in this study, including age range and sex distribution for young individuals, older individuals without parental longevity (OPUS), long‐lived individuals, and offspring of long‐lived individuals (OPEL). These data correspond to the groups described in Figure 1a.

**Table S2:** Nuclear proteome. Comprehensive list of nuclear proteins identified and quantified by mass spectrometry across all donor cohorts. The table includes protein identifiers, quantification values, and functional annotations. These data support the enrichment analysis presented in Figures 1d and 2a, highlighting succinylated proteins and pathways linked to chromatin organization and transcriptional regulation.

## Data Availability

All generated data are available as Supporting Information: tables in this manuscript.
