# Supplementary material for: Preliminary Evidence for Increased Histone Succinylation as a Potential Epigenetic Marker for Longevity
Source: Aging Cell. 2025 Dec 23;25(1):e70346. doi: 10.1111/acel.70346 (PMC12723716; doi:10.1111/acel.70346)
Supplement: Supplementary file 1 — Data S1: acel70346‐sup‐0001‐Supinfo.docx. [file ACEL-25-e70346-s003.docx]

**Supporting information**

**Preliminary Evidence for Increased Histone Succinylation as a Potential Epigenetic Marker for Longevity**

Stephanie Stransky^1α^, Sarah Graff^1α^, Kai Mao^2^, Derek M. Huffman^2^, Sofiya Milman^3,4^, Nir Barzilai^3,4^, Simone Sidoli^1*^

*^1^Department of Biochemistry, Albert Einstein College of Medicine, New York, NY, USA*

*^2^Department of Molecular Pharmacology, Albert Einstein College of Medicine, New York, NY, USA*

*^3^ Institute for Aging Research, Department of Medicine, Albert Einstein College of Medicine, New York, NY, USA*

*^4^ Department of Genetics, Albert Einstein College of Medicine, New York, NY, USA*

^α^ *Contributed equally to this work*

**Corresponding author:*

*Address: 1300 Morris Park Avenue, Bronx, NY 10461, USA*

*Telephone: +1 (718) 430-3475*

*E-mail address:* [*simone.sidoli@einsteinmed.edu*](mailto:simone.sidoli@einsteinmed.edu)

**Supplementary Table 1.** Information of the donor cohorts analyzed in this study, including age range and sex distribution for young individuals, older individuals without parental longevity (OPUS), long-lived individuals, and offspring of long-lived individuals (OPEL). These data correspond to the groups described in Figure 1a.

**Supplementary Table 2.** Nuclear proteome. Comprehensive list of nuclear proteins identified and quantified by mass spectrometry across all donor cohorts. The table includes protein identifiers, quantification values, and functional annotations. These data support the enrichment analysis presented in Figure 1d and Figure 2a, highlighting succinylated proteins and pathways linked to chromatin organization and transcriptional regulation.

**Materials and Methods**

**Blood collection and cell isolation**

B cells were isolated from blood samples collected from participants in the Longevity Genes Project, LonGenity, and LifeLong studies performed at Albert Einstein College of Medicine (Einstein) [1, 2]. All studies were approved by the Institutional Review Board (IRB) at Einstein under protocols 1998-125, 2007-272, and 2018-9258. The blood was drawn into BD Vacutainer tubes. After the Ficoll gradient centrifugation, peripheral blood mononuclear cells (PBMCs) were collected in 15ml centrifuge tube. According to the manufacturer’s protocol, PBMCs were mixed with CD19 microbeads (Miltenyi, 130-050-301), run through LS column, and washed, resulting in the release of the B cells from the column. Subsequently, the B cells were washed and frozen (~5 million cells/vail) by adding freezing media (90% culture media+10% DMSO). The cells were then transferred to a liquid nitrogen tank for long-term storage.

**Nuclear protein extraction and sample preparation**

The nuclear proteome was isolated from B cells of young, OPUS, OPEL and long-lived individuals. Briefly, the cell pellet was resuspended in five volumes of cold buffer A (10 mM ammonium bicarbonate pH 8, 1.5 mM MgCl_2_, and 10 mM KCl), incubated on ice for 10 min and centrifuged for 5 min at 400 g. The supernatant was removed, the pellet was resuspended in two volumes of buffer A containing 0.15% NP-40 and protease inhibitors and then centrifuged for 15 min at 3,200 g. The pellet was washed with ten volumes of PBS, centrifuged for 5 min at 3,200 g, and the supernatant was discarded. The pellet was resuspended with two volumes of buffer C (420mM NaCl, 20mM ammonium bicarbonate pH 8.0, 20% glycerol, 2 mM MgCl_2_, 0.2 mM EDTA, 0.1% NP-40, 10 mM sodium butyrate, 0.5 mM DTT, and complete protease inhibitors) and incubated for 1 h at 4ºC, on a rotating wheel. The suspension was centrifuged for 45 min at 20,800 g at 4ºC. The supernatant was stored at - 80ºC as the soluble nuclear fraction.

**Histone extraction and sample preparation**

Histone proteins were extracted from cell pellets, as described by [3]. Briefly, histones were acid extracted with cold 0.2 M sulfuric acid (5:1, sulfuric acid:pellet) and incubated for 2 h at 4ºC with constant rotation. Histones were then precipitated with 33% trichloroacetic acid (TCA) overnight at 4ºC. The supernatant was removed, tubes were rinsed with ice-cold acetone containing 0.1% HCl, centrifuged and rinsed again with100% ice-cold acetone. The supernatant was discarded, and the pellet was dried using the SpeedVac. Before derivatization, histones were resuspended in 50 mM ammonium bicarbonate (pH 8) containing 20% acetonitrile. In the fume hood, 2 µl of propionic anhydride and 10 µl of ammonium hydroxide (all Sigma Aldrich) were added to the samples. The mixture was incubated for 5 min and the procedure was repeated. Histones were then digested with 1 µg of sequencing grade trypsin (Promega) (1:20, enzyme:sample) diluted in 50 mM ammonium bicarbonate, pH 8, and incubated overnight at 37ºC. The derivatization reaction was repeated to derivatize peptide N-termini. The samples were dried in the Speedvac and stored in - 80ºC.

**Sample desalting**

Samples were desalted prior to mass spectrometry (MS) analysis as described by [3]. Briefly, samples were resuspended in 100 µl of 0.1% TFA and loaded into a 96-well filter plate (Orochem) packed with Oasis HLB C-18 resin (Waters), which was equilibrated using 100 µl of the same buffer. Samples were washed with 100 µl of 0.1% TFA, eluted with 70 µl of a buffer containing 60% acetonitrile and 0.1% TFA, and dried using SpeedVac.

**Histone post-translational modifications (PTMs) analysis**

After desalting, samples were resuspended in 10 µl of 0.1% TFA and loaded onto a Dionex RSLC Ultimate 300, coupled online with an Orbitrap Fusion Lumos (all Thermo Scientific). Chromatographic separation was performed using a two-column system, consisting of a C-18 trap cartridge (300 µm ID, 5 mm length) and an analytical column (75 µm ID, 25 cm length) packed in-house with reversed-phase Repro-Sil Pur C18-AQ 3 µm resin. Histone peptides were separated using a 60 min gradient from 4–30% buffer B (buffer A: 0.1% formic acid, buffer B: 80% acetonitrile + 0.1% formic acid) at a flow rate of 300 nl/min. The mass spectrometer was set to acquire spectra in a data-independent acquisition (DIA) mode. The full MS scan was set to 300–1100 m/z in the orbitrap with a resolution of 120,000 (at 200 m/z) and an AGC target of 5 × 105. MS/MS was performed in the orbitrap with sequential isolation windows of 50 m/z with an AGC target of 2 × 105 and an HCD collision energy of 30.

Raw files were imported into EpiProfile 2.0 software [4]. From the extracted ion chromatogram, the area under the curve was obtained and used to estimate the abundance of each peptide. To achieve the relative abundance of PTMs, the sum of all different modified forms of a histone peptide was considered as 100% and the area of the particular peptide was divided by the total area for that histone peptide in all of its modified forms. The relative ratio of two isobaric forms was estimated by averaging the ratio for each fragment ion with different mass between the two species. The resulting peptide lists generated by EpiProfile were exported to Microsoft Excel and further processed for a detailed analysis.

**Proteomics analysis**

After desalting, samples were resuspended in 10 µl of 0.1% TFA and loaded onto a Dionex RSLC Ultimate 300, coupled online with an Orbitrap Fusion Lumos (all Thermo Scientific). Chromatographic separation was performed using a two-column system, consisting of a C-18 trap cartridge (300 µm ID, 5 mm length) and an analytical column (75 µm ID, 25 cm length) packed in-house with reversed-phase Repro-Sil Pur C18-AQ 3 µm resin. Samples were separated using a 180 min gradient from 4 to 30% buffer B (buffer A: 0.1% formic acid, buffer B: 80% acetonitrile + 0.1% formic acid) at a flow rate of 300 nl/min. The mass spectrometer was set to acquire spectra in a data-dependent acquisition (DDA) mode. The full MS scan was set to 300–1200 m/z in the orbitrap with a resolution of 120,000 (at 200 m/z) and an AGC target of 5 × 10^5^. MS/MS was performed in the ion trap using the top speed mode (2 s), and AGC target of 1 × 10^4^ and an HCD collision energy of 35.

The MS raw data were processed using Proteome Discoverer software (v2.5, Thermo Scientific), SEQUEST search engine, and the SwissProt human database (updated June 2021). It was included variable modification of N-terminal acetylation and fixed modification of carbamidomethyl cysteine. Trypsin was specified as the digestive enzyme with two missed cleavages allowed. Mass tolerance was set to 10 ppm for precursor ions and 0.2 Da for product ions. Peptide and protein false discovery rate was set to 1%. Prior statistics, proteins were log2 transformed, normalized by the average value of each sample and missing values were imputed using a normal distribution 2 standard deviations lower than the mean as described [5]. Statistical regulation was assessed using heteroscedastic T-test (if p-value < 0.05). Data distribution was assumed to be normal, but this was not formally tested.

**Balance Beam Test**

Neuromuscular coordination was assessed using a balance beam test. Mice were first acclimated by walking across a flat plank (approximately 4 ft in length, 2 in wide) placed horizontally between two platforms, with a light source at the starting end and a food reward at the far end to encourage traversal. For testing, animals were challenged with three beams (1 in [easy], 0.75 in [medium], and 0.5 in [hard] diameter; 4 ft length) of increasing difficulty. Each mouse was placed on one end of the beam and allowed to walk to the opposite end. The number of total slips (defined as a foot misplacement off the beam) was recorded by direct observation [6, 7].

**Forelimb Grip Strength Test**

Forelimb grip strength was assessed using a weighted grasp assay. Each mouse was gently lifted and held by the tail, then presented with a 20 g weight attached to a wire hook. Mice instinctively grasped the hook with their forepaws, at which point the timer was started. Time to release the weight was then recorded over three attempts separated by a 3 min rest interval, and latency to release the weight calculated as the average of the two highest values [7].

**Frailty Index (FI)**

Physiological and functional frailty was evaluated using a validated 31-item mouse frailty index, as previously described (Whitehead et al., 2014). Briefly, mice were scored on multiple health-related domains, including integument (coat condition, alopecia), musculoskeletal system (kyphosis, grip strength), ocular and auditory function (cataracts, startle response), and general appearance/condition (body condition, piloerection, respiration). Each parameter was scored as 0 (no deficit), 0.5 (mild deficit), or 1 (severe deficit). The FI score for each animal was calculated as the sum of observed deficits divided by the total number of items assessed, yielding a value between 0 (no frailty) and 1 (maximal frailty) [7, 8].

**Box Maze**

Mice were placed individually in a brightly illuminated square arena containing seven false escape routes and one true exit leading to a dark, enclosed box. Each animal first underwent a 2-minute trial session in which visual cues were provided and the true escape location was demonstrated to establish spatial memory. This was followed by four consecutive test sessions, each lasting up to 10 minutes and separated by 2-minute rest intervals. During testing, the latency to locate the true escape and the number of false exit attempts were recorded [7, 9].

**References:**

1. Gubbi, S., et al., *Effect of Exceptional Parental Longevity and Lifestyle Factors on Prevalence of Cardiovascular Disease in Offspring.* Am J Cardiol, 2017. **120**(12): p. 2170-2175.

2. Barzilai, N., et al., *Unique lipoprotein phenotype and genotype associated with exceptional longevity.* JAMA, 2003. **290**(15): p. 2030-40.

3. Stransky, S., et al., *Investigation of reversible histone acetylation and dynamics in gene expression regulation using 3D liver spheroid model.* Epigenetics Chromatin, 2022. **15**(1): p. 35.

4. Yuan, Z.F., et al., *EpiProfile 2.0: A Computational Platform for Processing Epi-Proteomics Mass Spectrometry Data.* J Proteome Res, 2018. **17**(7): p. 2533-2541.

5. Aguilan, J.T., K. Kulej, and S. Sidoli, *Guide for protein fold change and p-value calculation for non-experts in proteomics.* Mol Omics, 2020. **16**(6): p. 573-582.

6. Mao, K., et al., *Late-life targeting of the IGF-1 receptor improves healthspan and lifespan in female mice.* Nat Commun, 2018. **9**(1): p. 2394.

7. Mao, K., et al., *Salutary effects of transdermal curcumin on multiple indices of health span in rodent models of normal aging and hypertension.* Geroscience, 2025. **47**(4): p. 5577-5590.

8. Whitehead, J.C., et al., *A clinical frailty index in aging mice: comparisons with frailty index data in humans.* J Gerontol A Biol Sci Med Sci, 2014. **69**(6): p. 621-32.

9. Engel, M.G., et al., *Intranasal long R3 insulin-like growth factor-1 treatment promotes amyloid plaque remodeling in cerebral cortex but fails to preserve cognitive function in male 5XFAD mice.* J Alzheimers Dis, 2025. **103**(1): p. 113-126.
